# Synergistic High Charge-Storage Capacity for Multi-level Flexible Organic Flash Memory

**DOI:** 10.1038/srep12299

**Published:** 2015-07-23

**Authors:** Minji Kang, Dongyoon Khim, Won-Tae Park, Jihong Kim, Juhwan Kim, Yong-Young Noh, Kang-Jun Baeg, Dong-Yu Kim

**Affiliations:** 1 Heeger Center for Advanced Materials, School of Materials Science and Engineering, Gwangju Institute of Science and Technology (GIST), 261 Cheomdan-gwagiro, Buk-gu, Gwangju 500-712, Republic of Korea; 2 Department of Physics, Blackett Laboratory, Imperial College London, London, SW7 2AZ, UK; 3 Department of Energy and Materials Engineering, Dongguk University, 26 Pil-dong, 3-ga, Jung-gu, Seoul 100-715, Republic of Korea; 4 Department of Chemical Engineering and Materials Science, University of California, Irvine, Irvine, California 92697, United States; 5 Nanocarbon Materials Research Group, Korea Electrotechnology Research Institute (KERI) 12 Bulmosan-ro 10Beon-gil, Seongsan-gu, Changwon, Gyeongsangnam-do 642-120, Republic of Korea

## Abstract

Electret and organic floating-gate memories are next-generation flash storage mediums for printed organic complementary circuits. While each flash memory can be easily fabricated using solution processes on flexible plastic substrates, promising their potential for on-chip memory organization is limited by unreliable bit operation and high write loads. We here report that new architecture could improve the overall performance of organic memory, and especially meet high storage for multi-level operation. Our concept depends on synergistic effect of electrical characterization in combination with a polymer electret (poly(2-vinyl naphthalene) (PVN)) and metal nanoparticles (Copper). It is distinguished from mostly organic nano-floating-gate memories by using the electret dielectric instead of general tunneling dielectric for additional charge storage. The uniform stacking of organic layers including various dielectrics and poly(3-hexylthiophene) (P3HT) as an organic semiconductor, followed by thin-film coating using orthogonal solvents, greatly improve device precision despite easy and fast manufacture. Poly(vinylidene fluoride-trifluoroethylene) [P(VDF-TrFE)] as high-k blocking dielectric also allows reduction of programming voltage. The reported synergistic organic memory devices represent low power consumption, high cycle endurance, high thermal stability and suitable retention time, compared to electret and organic nano-floating-gate memory devices.

Recently, soaring interests in wearable smart devices have stirred up the development of electronically functional new materials and devices with mechanically flexible/stretchable properties. Currently, accessary-type wearable devices such as smart watches and glasses are expected to further progress into an electronic system on human skin (e-skin) and in clothes (e-textile)^1^,^2^,^3^. A number of research activities have been conducted to develop these soft electronic (transistors and logics)^4^,^5^,^6^, optoelectronic (light-emitting diodes and photosensors)^7^,^8^,^9^,^10^, and energy (photovoltaics, batteries, and nano-generators) devices^11^,^12^,^13^,^14^ for these applications^15^,^16^. Wearable solid-state data storage is also needed for storage of an operating system and for personalized information. The memory element should be mechanically flexible (even stretchable), cost-effective, and electrically programmable and erasable^17^. In addition, it must have the capability for stable data retention, fast switching time, low-power operation, and high storage^18^,^19^. Because current inorganic-based memory devices are clearly incompatible with flexible substrates and textiles, non-volatile organic memory based on π-conjugated molecules is considered as a promising candidate to fulfil these requirements^19^,^20^,^21^,^22^. Moreover, the functionality of these materials can be simply tuned by engineering of the molecular design and synthesis; thus, they possess versatile processability via graphic art printing processes^23^,^24^.

Although remarkable progress has been achieved over the last decade, organic flash memory is still not adequate for practical application in flexible and wearable smart devices mainly due to their relatively short retention time, high operating bias, limited endurance during device operation, and small data-storage capacity per unit area^25^,^26^. The relatively low patterning resolution of solution-based common printing processes (>30 μm) limits the data-storage capacity^26^. To increase memory capacity, high-resolution patterning processes such as self-aligned inkjet printing, electro-hydrodynamic jetting, and nanoimprint lithography should be applied^27^,^28^. Alternatively, the capacity can also be remarkably increased by evolution of the multi-level memory characteristics. Most organic memory devices simply use two different electrical states, namely, high (‘ON’) and low (‘OFF’) conductivity states, which store binary data (‘0’ or ‘1’). On the other hand, if we can access many intermediate electrical states between the ON and OFF states, multi-level storage of more than ternary data in the same device area can possibly be achieved^29^,^30^.

To realize high density and reliable data storage in a multi-level memory, large stored-charge density (Q_t_) and precise linear control of the number of trapped charge carriers by applying external electric fields are required^31^. For a transistor memory device, conductance between the source and drain electrodes is controlled by electric field modulation of the gate electrode either by trapping charge carriers in the gate dielectrics, such as chargeable dielectrics (electrets) and nano-floating gates (NFGs), or by electric-field-induced and permanent dipoles using ferroelectric materials^24^,^25^,^30^,^32^,^33^,^34^. To realize a multi-level transistor memory, therefore, we should increase the stored charge carrier density in the gate dielectric layers (which depend on the number of trap sites and trapped charges) and linearly control the threshold voltage (V_Th_) by applying different programming and erasing biases. These discrete electrical states can then be read out by typically applying a reading bias at zero gate bias. Thus, this technology can be a fundamental building block for high-density flexible/printed organic flash memory. In addition, it can be applied for non-destructive read-out without biasing the gate field, and it possesses excellent process compatibility with peripheral complementary metal oxide semiconductor circuitry to address each memory cell. Few studies have been conducted to realize multi-level organic transistor memory devices using ferroelectric Poly(vinylidene fluoride-trifluoroethylene) [P(VDF-TrFE)]^32^ or an ambipolar polymer semiconductor^35^. However, systematic study to realize multi-level organic transistor memory has been rare.

In the current study, we propose a method to remarkably increase the memory capacity of printed/flexible organic flash memory via synergistic charge storages composed of chargeable organic materials (electrets) and NFGs for simultaneous charge trapping at both sites of a single-transistor memory device. The two different charge-storage sites, i.e. polymer electrets and copper (Cu) nanoparticle (NP)-based NFGs, in the proposed organic memory devices can effectively increase the number of charge trap sites and control the charge trapping and linear erasing using external electric field. The dual charge trapping sites, referred to here as synergistic memory, provide significantly improved non-volatile memory characteristics compared with common organic transistor memories with a single charge trapping site of either electrets or NFG memories (NFGMs). These characteristics include a wide memory window of ~42.6 V (almost 85.2% of the applied bias), linear shifts in V_Th_ under various gate bias conditions, a multi-level (nine levels per cell) data storage, very reproducible memory cycling endurance during repeated write–read–erase processes (over 100 times), an excellent stability for mechanical bending stress of over 1000 times, and quasi-permanent data retention characteristics (>10^8^ s).

## Results and Discussion

**Figure 1:**
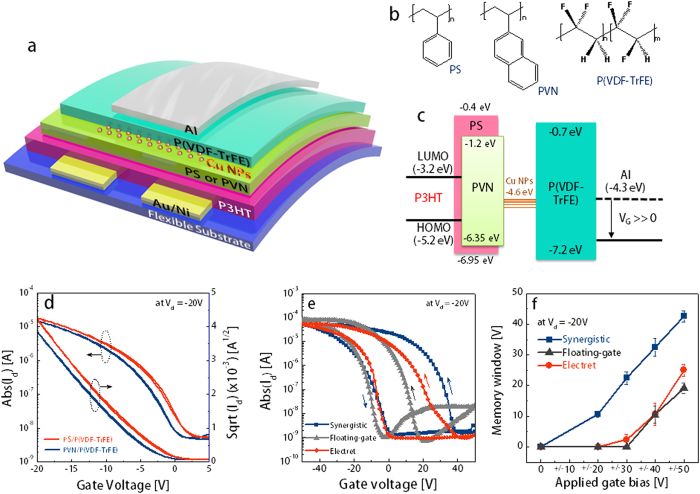
(**a**) Schematic of a flexible organic transistor memory device based on dual charge-storage media. (**b**) Chemical structures of P3HT, PS, PVN, and P(VDF-TrFE) (**c**) Energy diagram of OFET-based memories. (**d**) Initial transfer characteristics (I_d_ *versus* V_g_) of P3HT OFETs with bi-layered polymer dielectrics: PS/P(VDF-TrFE) and PVN/P(VDF-TrFE) [*W/L* = 1 mm/10 μm]. (**e**) Memory characteristics of various memory device modes: electret [PVN/P(VDF-TrFE)], NFG [PS/Cu NPs/P(VDF-TrFE)], and synergistic [PVN/Cu NPs/P(VDF-TrFE)] memories, where the transfer plots are obtained by bi-directional V_g_ sweeps from +50 to −50 V at V_d_ = −20 V. (**f**) Memory windows of the corresponding memory devices at different ranges of applied gate biases from ±20 to ±50 V.

Figure 1a,b show the schematic of a flexible organic transistor memory with dual memory elements using electrets and NFGs and the chemical structures of the organic semiconductor (P3HT) and dielectrics: polystyrene (PS), poly(2-vinyl naphthalene) (PVN), and P(VDF-TrFE). A typical ferroelectric polymer P(VDF-TrFE) is used here as a relaxor high-*k* (*k* is the dielectric constant = ~10.5) dielectric layer without thermal annealing for low-voltage operation^33^. This layer is also used in a low-*k*/high-*k* bi-layered dielectric system to induce an effective charge injection into the charge trap sites because a higher electric field is mainly loaded in the low-*k* dielectric layer^36^. Thus, PS (*k* ≈ 2.45)/P(VDF-TrFE) and PVN (*k* ≈ 2.65)/P(VDF-TrFE) solutions are sequentially deposited using orthogonal solvents. PS (~60 nm thick) and PVN (~70 nm thick) are used as follows: (*i*) passivat*i*on layer of a semiconductor active channel to prevent penetration of metal NPs during thermal evaporation, (*ii*) tunnelling dielectric layer for effective charge carrier injection via Fowler–Nordheim tunnelling, thus preventing loss of stored charge carriers, and (*iii*) polymer electret layer for storage of additional self-electrical charging. We note that PVN shows an excellent charge-storing efficiency compared with PS owing to its more extended π-conjugation^37^. Figure 1c shows that PVN has a smaller energy barrier for electron injection (~2.0 eV) from P3HT than PS (~2.8 eV). Therefore, the injected charges from the transistor channel are mostly trapped in the electret of the PVN/P(VDF-TrFE) devices^33^, whereas they are mostly trapped in Cu NPs of the PS/P(VDF-TrFE) devices.

Figure 1d shows the initial transfer characteristics [drain current (I_d_) *versus* gate voltage (V_g_)] of the P3HT organic field-effect transistors (OFETs) at a drain voltage (V_d_) of −20 V with bi-layered polymer dielectrics: PS/P(VDF-TrFE) and PVN/P(VDF-TrFE) [channel width/length *W/L* = 1 mm/10 μm]. All P3HT OFETs show reasonable hole mobilities of ~0.05 cm^2^·V^−1^·s^−1^ without any hysteresis. The basic device parameters are listed in Table 1. To compare the electrical characteristics of the various OFET memory device modes, three different gate dielectric structures were manufactured: (*i*) electrets-only memory with PVN/P(VDF-TrFE), (*ii*) NFGM with PS/Cu NPs/P(VDF-TrFE), and (*iii*) synergistic memory (electret + NFGM) with PVN/Cu NPs/P(VDF-TrFE). Figure 1e shows that all memory devices displayed remarkable anti-clockwise hysteresis loops between the forward and reverse gate bias sweeps. The counter-clockwise hysteresis feature reveals that these memory devices mostly operate by trapping and detrapping the charge carriers in the gate dielectrics. Thus, at the programmed state (ON state), the memory devices are set up with a positive gate bias that transfers mobile electrons from the OFET channel to the trapping sites, whereas in the erased state (OFF state), the stored electrons in the trap sites are detrapped under a negative gate bias.

In addition, we used the P3HT as an active layer because it has been used as a very typical p-type organic semiconducting material. To more generalize our synergistic memory characteristics, we applied with various material collections, different classes of high-performance organic semiconductors (OSC) (p-type OSC: dodecyl substituted thienylenevinylene-thiophene copolymer (PC12TV12T) and ambipolar OSC: diketopyrrolopyrrolethieno[3,2-b]thiophene copolymer (DPPT-TT)) instead of the P3HT (Figure S1). In comparison to individual NFG and electret memory devices, synergistic memory devices based on both PC12TV12T and DPPT-TT also showed much larger memory window (width of I_D_-V_G_ hysteresis) and lower operating voltage (see Figure S1a and S1b). Furthermore, to clarify the PC12TV12T-based synergistic memory devices, the representative programming and erasing characteristics were measured, as shown in Figure S1c and S1d. The memory devices showed a high charge trap density and stable memory cycle endurance similar to P3HT-based memory devices.

The maximum width of a hysteresis loop refers to the memory window (ΔV_Th_), which is a representative figure-of-merit for transistor-type memory devices. The memory windows are obtained by changing the V_g_ sweep ranges from ±20 to ±50 V (please see Fig. 1f). Notably, single-mode memory devices, either electret memory or NFGM, exhibit distinctive memory windows when high V_g_ of more than ±40 V is applied. After the application of large forward and reverse V_g_ sweeps from +50 to −50 V, the electret memory (with PVN electret) and NFGM (with Cu NPs) devices show memory windows of ~25 V (which is approximately 50% of the applied bias) and ~19 V (~38% of the applied bias), respectively. The memory window, which is determined by ΔV_Th_ = −Q_t_/C_i_, should be large enough to distinguish different electrical memory states to achieve more multi-level memory states than the binary ON and OFF states. Decreasing C_i_ to _i_mprove the memory window is not a good strategy in practical applications because small C_i_ inev_i_tably requires very high operating bias. Thus, we should increase the stored charge carrier density Q_t_ mainly by increasing the number of trap sites. The maximum Q_t_ values of the electret memory and NFGM devices are limited to ~2.4 × 10^12^ and ~2.8 × 10^12^ cm^−2^, respectively. To increase Q_t_, we used dual charge trapping sites, which consist of organic electrets and metallic NFGs. As additional charge trap sites, Cu NPs were incorporated by thermal evaporation (deposition thickness: t_NP_ ≈ 1.0 nm; please see supporting information Figure S2, S3) between the PVN/P(VDF-TrFE) bi-layered dielectrics. Impressively, the synergistic memory devices showed much larger memory windows (of ~43 V) than the electret memory (~25 V) and NFGM (~19 V) devices under the same applied bias range conditions (at V_g_ = ±50 V). Such large memory window is attributed to the increase in the charge density stored in the charge trap elements, i.e. both the Cu NFG and PVN electret layers. The synergistic memory device exhibited an almost two times larger Q_t_ value of ~4.8 × 10^12^ cm^−2^ than the device with a single trapping site (NFGM or electret memory). This result reveals that our approach using dual trapping sites effectively increases Q_t_ and is a promising strategy for realizing multi-level organic flash memory in terms of large charge-storage capacity. In Fig. 1f, more than 40 memory devices have took into consideration to measure the memory window for each memory devices; electret, nano-floating-gate, and synergistic memories, where the average memory window (standard deviation) was ~25.0 V (±1.9 V), ~19 V (±1.7 V), and ~42.6 V (±1.9 V), respectively. Note that all memory configurations exhibited relatively uniform and repeatable switching characteristics between the ON- and OFF-states.

**Figure 2:**
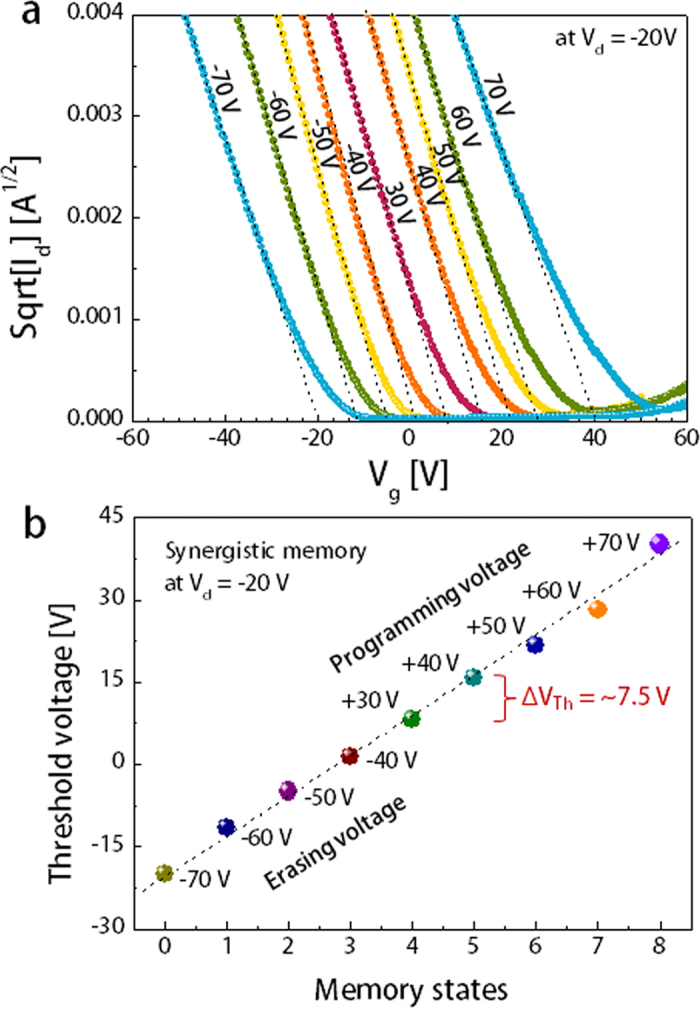
Multi-level data storage in synergistic memory devices. (**a**) Linearly shifted transfer plots (square root of I_d_ *versus* V_g_) by applying various programming and erasing biases. (**b**) Corresponding V_Th_ values at each well-defined data level with a V_Th_ interval of 7.5 V.

Figure 2a,b show the linear shifts in the transfer plots (square root of I_d_ *versus* V_g_) and the corresponding V_Th_ values in a synergistic memory device under different applied biases. By changing the programming voltages (from +30 to +70 V) and erasing voltages (from −40 to −70 V), the V_Th_ value linearly changed from −18 to +41 V. We obtained approximately nine stages for each data storage levels, and the devices exhibited a linear trend with ~7.5-V V_Th_ variation (Fig. 2b). The controlla**b**le V_Th_ implemented various current levels to detect the electrical memory states, which revealed that the amount of charge carriers stored in the trap sites could be reversibly modulated by applying external gate fields. Cu NPs were spatially distributed with slightly different sizes between the electret and blocking dielectric layer (see Figure S2), which may form discrete charge storing sites with different energy barrier to trap the mobile charge carriers. Energy levels of trap sites in the electrets also presumably have Gaussian distribution. Therefore, the number of accessible trapping sites is gradually increased by increasing the programming gate bias. These enhanced multi-level memory states are mostly enabled by increasing the trapped charge density in both electret and NFGs, leading to a relatively large memory window that separates the discrete memory states. The well-defined current states, obtained from the enough data margins, could easily allow to increase write precision within available access time and store more data than 1 bit on a single memory cell. Notably, the programmable gate bias of the synergistic memory is two times lower (higher than ±20 V) than that of the individual electret memory and NFGM, which is ±40 V. Although it is difficult to completely understand which site more predominately traps the electrons at each programming and erasing voltage, charge trapping process is presumed to simultaneously occur in the electrets and NFGs, resulting in relatively lower operating voltages.

**Figure 3:**
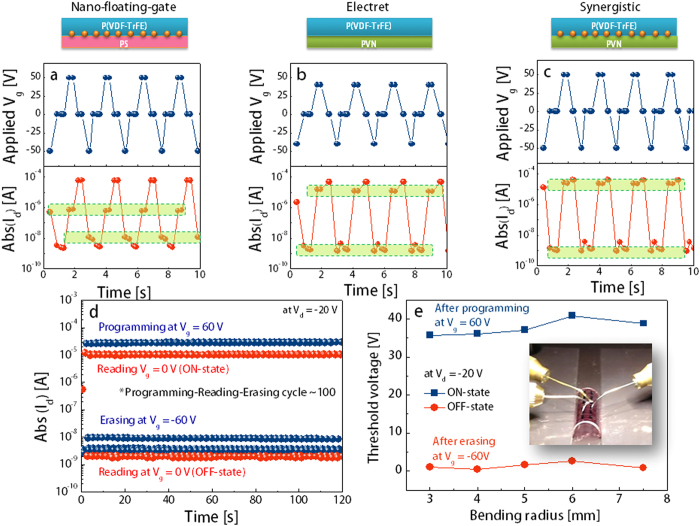
Reliable memory characteristics of various organic memory device structures. Programming–reading–erasing memory cycling endurance for (**a**) NFGM, (**b**) electret memory, and (**c**) synergistic memory devices on a glass substrate. (**d**) The same memory characteristics of the synergistic memory on a flexible PEN substrate. (**e**) V_Th_ shifts of the flexible synergistic memory device after programming and erasing at different bending radii (inset: photograph of the flexible synergistic memory device).

Figure 3a,c show the memory cyclic endurance and corresponding cross-sectional images of various organic memory devices according to the combination of bi-layered dielectrics and NFGs. Multiple memory cycles, i.e. programming–reading–erasing processes, were obtained by measuring I_d_ at V_d_ = −20 V using sequential application of the voltages for programming (V_g_ = +50 V for NFGM and synergistic memory; V_g_ = +40 V for electret memory), reading (V_g_ = 0 V), and erasing (V_g_ = −50 V for NFGM and synergistic memory; V_g_ = −40 V for electret memory). A positive voltage bias applied for ~0.1 s induced accumulation of electrons near the semiconductor–dielectric layer interface and transferred electrons into the charge trap sites. The trapped electrons were preserved and caused the high-I_d_ (ON) state to remain even though V_g_ returned to zero. On the other hand, when a negative voltage pulse was applied for ~0.1 s, the stored electrons were detrapped mainly by being compensated with the transferred positive charge carriers (holes) from the active channel. Then, the devices changed to their low-I_d_ (OFF) state. Figure S4 shows that all memory configurations exhibited reproducible and reversible switching between the ON and OFF states over at least 100 cycles within a relatively short time scale. Obviously, the synergistic memory exhibited the largest ON/OFF ratio of ~10^5^, faster switching time, and excellent cyclic endurance compared with the other memory devices.

The flexible memory devices with dual trap sites on the polyethylene naphthalate (PEN) substrate also showed excellent reliability and reproducibility with a well-defined programmed and erased states for more than 100 cycles without any degradation in the electrical characteristics (please see Fig. 3d). The electrical stability of the flexible memory devices was tested under repeated bending-stress conditions, as shown in Fig. 3e. The charg**e** carrier mobility retained almost its initial value when the bending radius was reduced from 6 to 3 mm. We note that the measured V_Th_ values after programming (V_g_ = 60 V) and erasing (V_g_ = −60 V) slightly changed at each bending radius. Moreover, the memory characteristics remained stable during continuous mechanical stress (please see cycling test results in Figure S5), which could be attributed to the soft mechanical properties of polymer semiconductors and all charge-storage sites in bi-layered polymer gate dielectrics. These mechanical and electrical stabilities prove that our synergistic memory is a reliable data storage, which satisfies practical printed/flexible electronic applications, and works well without any degradation during and/or after mechanical bending.

**Figure 4:**
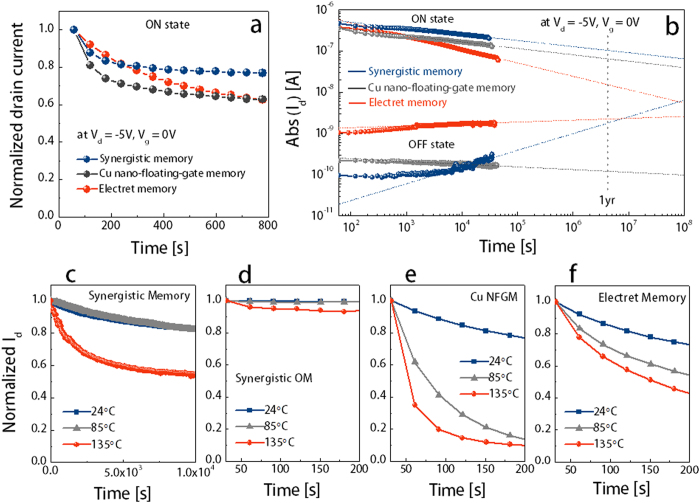
Retention characteristics of various organic transistor memory devices, namely, electret, Cu NFG, and synergistic memories. (**a**) Normalized drain current at the initial charge-relaxation stage. (**b**) Evolution of the charge relaxation with extended time scale to estimate the retention time in which the ON and OFF state currents were measured at V_d_ = −5 V and V_g_ = 0 V at a time interval of 60 s after application of programming (V_g_ = +50 V) and erasing (V_g_ = −50 V) biases for 1 s. (**c**)–(**f**) Normalized drain current at the programmed state as a function of time under different temperatures.

Figure 4 shows the retention characteristics of the electret memory, NFGM, and synergistic memory devices in which the programmed (ON) and erased (OFF) states were obtained after application of a gate bias of +50 and −50 V for 1 s, respectively. Interestingly, the three memory devices showed quite different charge-relaxation characteristics. Compared with the NFGM that used a PS tunnelling layer (showing a small electret effect), the electret and synergistic memory devices with a PVN electret/tunnelling layer exhibited slow initial charge relaxation, as shown in Fig. 4a. Although the NFGM showed relatively fast initial decay, the longest retention characteristics were obtained from the Cu NP-embedded memory devices such as the NFGM and synergistic memory devices (please see Fig. 4b). The stored charge loss typically depends on the following: 1) localized energy states that provide leakage pathways through the tunnelling and blocking dielectric layers, 2) energy levels of the charge trap sites and its barrier offset with the tunnelling layer, 3) polarity of the electret materials, 4) size and distribution of metallic NPs, and 5) film thickness and quality of the tunnelling and charge blocking dielectrics^34^,^37^,^38^. Moreover, organic transistor memory generally has two different charge-relaxation regimes, i.e. initial exponential fast decay followed by steady slow relaxation. Initial fast decay may occur when the trapped charge is easily compensated by nearby counter charge carriers within the charge migration/diffusion length^39^. After the nearby counter charges within the diffusion length are exhausted, the trapped charges could be dissipated by the counter charge carriers generated by slow thermal excitation. Although the exact charge-relaxation mechanism is not completely understood, we believe that the relatively fast initial decay of NFGM occurs because Cu NPs have higher electrostatic potential energy than the trapped individual electrons in the electrets, thus strongly attracting the nearby counter charge carriers. However, a much deeper charge-storage wall and a stronger charge confinement in the Cu NPs resulted in a very long charge retention time. Obviously, the Cu NPs played a key role in ensuring long-term retention of synergistic memory devices. The slightly faster charge dissipation in the dual trap site devices is attributed to a smaller energy band offsfet between the Cu NPs and conduction band of the PVN than that between the Cu NPs and PS layer^40^. Nevertheless, the synergistic memory also showed quasi-permanent retention time of more than 10^8^ s, estimated by extrapolation of ON and OFF state I_d_ over an extended time scale (please see Fig. 4b). This retention time is much longer than those of the other OFET-based memory devices and enough to satisfy the requirement for state-of-the-art solid-state data storage of 10 years.

The synergistic memory showed a better charge retention stability at high operating temperature. Figure 4c–f show that I_d_ of the NFGM, electrets, and synergistic memory after programming slowly decreased at room temperature, whereas at high temperature (85 and 135 °C), the increased thermal energy accelerated the decay of I_d_ due to the thermally activated charge carriers in the charge trap sites^41^. Notably, the ON current of the electret memory decayed more slowly at high temperature than that of the NFGM device. Although, more studies are still required, this retention characteristics is mainly attributed to the fact that the PVN electret has higher thermal stability to store charge carriers than the Cu NFG owing to its excellent electrical insulating and mechanical features with higher glass transition temperature T_g_ of ~135 °C than T_g_ of PS, which is ~95 °C^42^,^43^. For most polymeric materials, thermal distortions sharply occur above T_g_; thus, the NFGM device exhibited worse thermal stability because of the lower T_g_ of the PS film than that of the PVN film^44^. Impressively, our dual charge trap site memory devices showed excellent charge retention characteristics even at high temperature (Fig. 4d) via the synergistic charge-storing effects in the electret and NFG.

## Conclusions

In conclusion, we have successfully demonstrated a printed/flexible organic transistor memory with synergistic charge-storage media combination of electrets and metallic NFGs. This structure provides a simple and powerful concept for ultra-stable OFET-based non-volatile memory with dramatically improved charge-storage capacity as a framework for multi-level organic flash memory of as high as nine levels per cell. The high reliability and increased memory density are mostly attributed to the synergistic effect of the NFGs as ‘quasi-permanent’ charge trapping sites and to the efficiently chargeable polymer electrets as a tunnelling dielectric layer. Plastic synergistic memory devices also show reliable memory performance under high temperature and high bending condition at a radius of ~3 mm. We expect that our organic flash memory could be used in manufacturing high-density stable data storage in various wearable electronic devices.

## Methods

### FET Fabrication

The device structure of the top-gate/bottom-contact OFET-type memory is schematically shown in Fig. 1a. Corning XG glass or PEN film with Au/Ni (15 nm/5 nm thick) and patterned S/D electrodes using conventional photolithography were used as substrates. After ultraviolet–ozone treatment of the cleaned substrates, a P3HT solution dissolved in chlorobenzene (10 mg/ml) was spin-coated at 2000 rpm for 60 s. The P3HT film was then thermally annealed in a N_2_-purged glove box at 150 °C for 30 min. For the fabrication of the bi-layered dielectric film, PS and PVN were dissolved in n-butyl acetate and 2-butanone (5–10 mg/ml), respectively. P(VDF-TrFE) (65:35 mol%) was dissolved in acetonitrile (30–40 mg/ml). The solutions were sequentially spin coated at 2000 rpm for 60 s. The drying conditions for the PS (or PVN) and P(VDF-TrFE) films were 80 °C for 30 min and 60 °C for 10 min (or no thermal annealing), respectively. For the NFGM and SM devices, Cu NPs were prepared by thermal evaporation on PS and PVN before deposition of the P(VDF-TrFE) films. Finally, aluminium gate electrodes (~45 nm) were thermally evaporated onto the dielectric layers, patterned with a metal shadow mask.

### Characterisation

The electrical characteristics of the devices were measured using a Keithley 4200-SCS in a N_2_-purged glove box. The dielectric film thicknesses were measured using an XP-1 surface profiling system (Ambios Technology, Inc.). Transmission electron microscope (TEM) images were taken using a Technai G2 S-Twin field-emission TEM operating at 200 keV. Atomic force microscopy (AFM) experiments were performed using a Digital Instruments Multimode atomic force microscope controlled by a Nanoscope IIIa scanning probe microscope controller. Chemical composition of the surface was characterized using an AXIS – NOVA (KRATOS Inc.) X-ray photoelectron spectrometer (XPS) with a scanning monochromatic Al source (1486.6 eV). The surface morphology of the films was imaged using a tapping-mode atomic force microscope (Nanoscope III, Veeco Instruments, Inc.) at the Korea Basic Science Institute (KBSI).

## Additional Information

**How to cite this article**: Kang, M. *et al.* Synergistic High Charge-Storage Capacity for Multi-level Flexible Organic Flash Memory. *Sci. Rep.* **5**, 12299; doi: 10.1038/srep12299 (2015).

## Supplementary Material

Supplementary Information

## Figures and Tables

**Table 1:** **Fundamental parameters of the P3HT-based OFETs with two types of gate dielectrics**.

**Parameters**	**PS/P(VDF-TrFE)**	**PVN/P(VDF-TrFE)**
Dielectric constant	2.45/10.5	2.65/10.5
Thickness [nm]	~60/215	~70/210
Capacitance [nF/cm^2^]	20	18
Mobility [cm^2^/Vs] (at V_d_ = −20)	0.050 ± 0.010	0.052 ± 0.012
I_on_/I_off_	3 × 10^3^	5 × 10^3^
V_Th,i_ [V]	−4.65 ± 0.66	−4.03 ± 0.20
E_i_ [MV/cm]	6.3	5.9
