# Supplementary material for: Synergistic High Charge-Storage Capacity for Multi-level Flexible Organic Flash Memory
Source: Sci Rep. 2015 Jul 23;5:12299. doi: 10.1038/srep12299 (PMC4511867; doi:10.1038/srep12299)
Supplement: Supplementary Information [file srep12299-s1.doc]

Supporting Information

Synergistic High Charge-Storage Capacity for Multi-level Flexible Organic Flash Memory

*Minji Kang^1^, Dongyoon Khim^2^, Won-Tae Park^3^, Jihong Kim^1^, Juhwan Kim^4^, Yong-Young No^3^, Kang-Jun Baeg^5^, Dong-Yu Kim^1^*

^1^Heeger Center for Advanced Materials, School of Materials Science and Engineering, Gwangju Institute of Science and Technology (GIST), 261 Cheomdan-gwagiro, Buk-gu, Gwangju 500-712, Republic of Korea, ^2^Department of Physics, Blackett Laboratory, Imperial College London, London, SW7 2AZ, UK, ^3^Department of Energy and Materials Engineering, Dongguk University, 26 Pil-dong, 3-ga, Jung-gu, Seoul 100-715, Republic of Korea, Department of Chemical Engineering and Materials Science, ^4^University of California, Irvine, Irvine, California 92697, United States, ^5^Nanocarbon Materials Research Group, Korea Electrotechnology Research Institute (KERI) 12 Bulmosan-ro 10Beon-gil, Seongsan-gu, Changwon, Gyeongsangnam-do 642-120, Republic of Korea.

Correspondence and requests for materials should be addressed to Y.Y.N. (yynoh@dongguk.edu) or K.J.B (kangjun100@keri.re.kr) or D.Y.K. (kimdy@gist.ac.kr)

Keywords: organic non-volatile memory, electret, floating gate, organic field-effect transistor, conjugated polymers


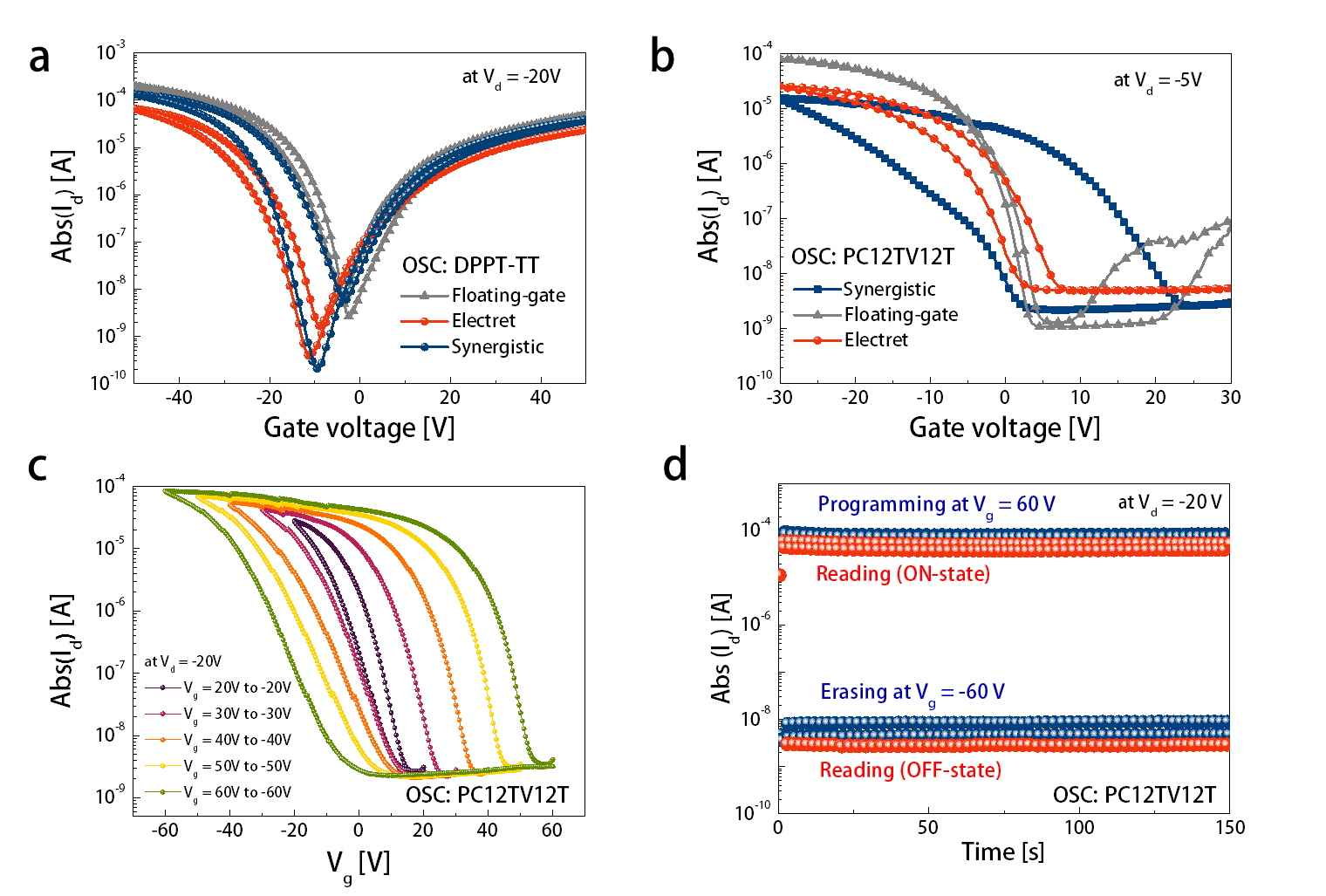


**Figure S1.** Memory characteristics of synergistic memory devices based on a) DPPT-TT and b) PC12TV12T. c) Transfer plots (I_D_-V_G_) by applying various programming and erasing biases and d) switching test over at least 100 cycles in PC12TV12T-based synergistic memory devices.


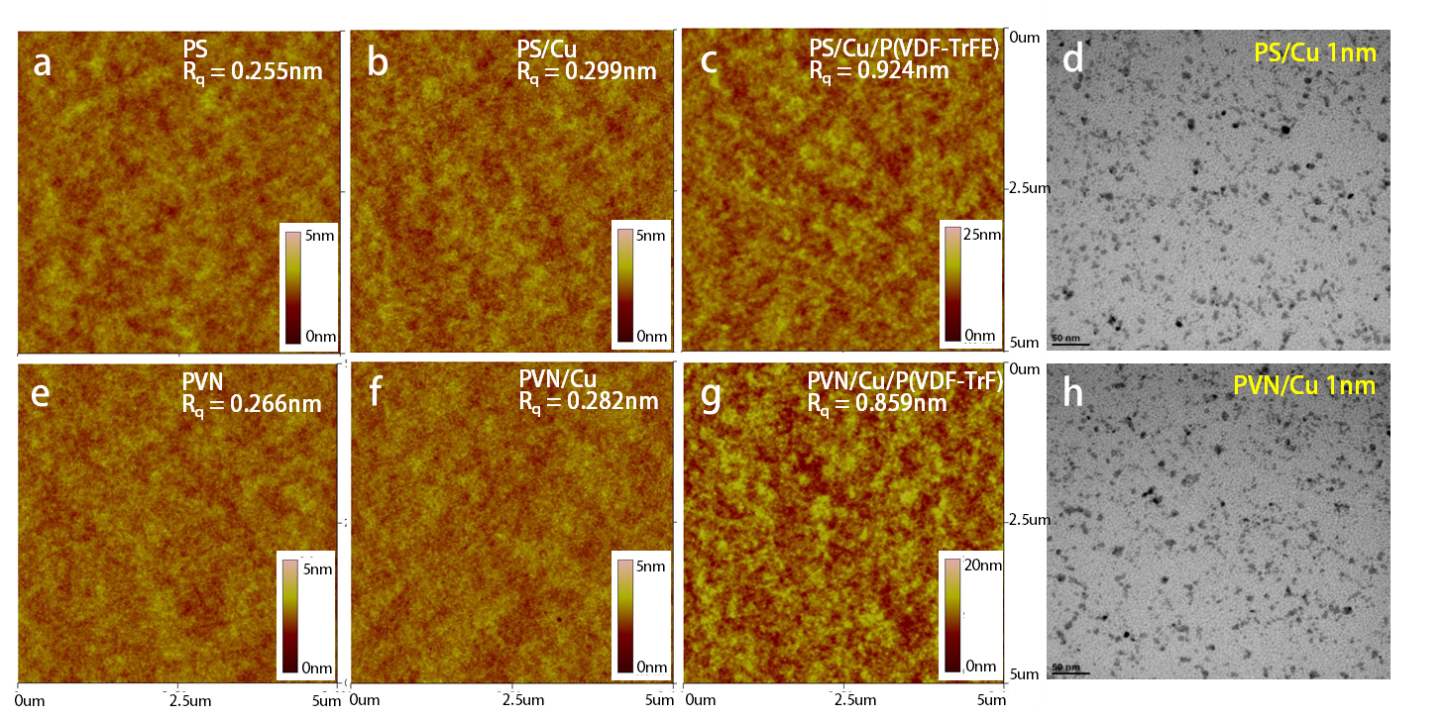


**Figure S2. Atomic force microscopy (**AFM) images of various dielectric layers w/ or w/o Cu nanoparticles (NPs) in height mode. a) PS single layer, b) Cu NPs on PS, C) PS/P(VDF-TrFE) bilayer embedded Cu NPs, e) PVN single layer, f) Cu NPs on PVN, g) PVN/P(VDF-TrFE) bilayer embedded Cu NPs. TEM images of thermally deposited Cu NPs on d) PS and h) PVN.

a

b

**Figure S3. X-ray photoelectron spectroscopy (XPS) analyses for dielectric layers w/ or w/o Cu NPs.** a) The survey scan and b) the Cu 2p core level binding energy spectra represent two peaks located at 932.1 eV and 952.1 eV, which corresponds to the Cu 2p_3/2_ and Cu 2p_1/2_, respectively.


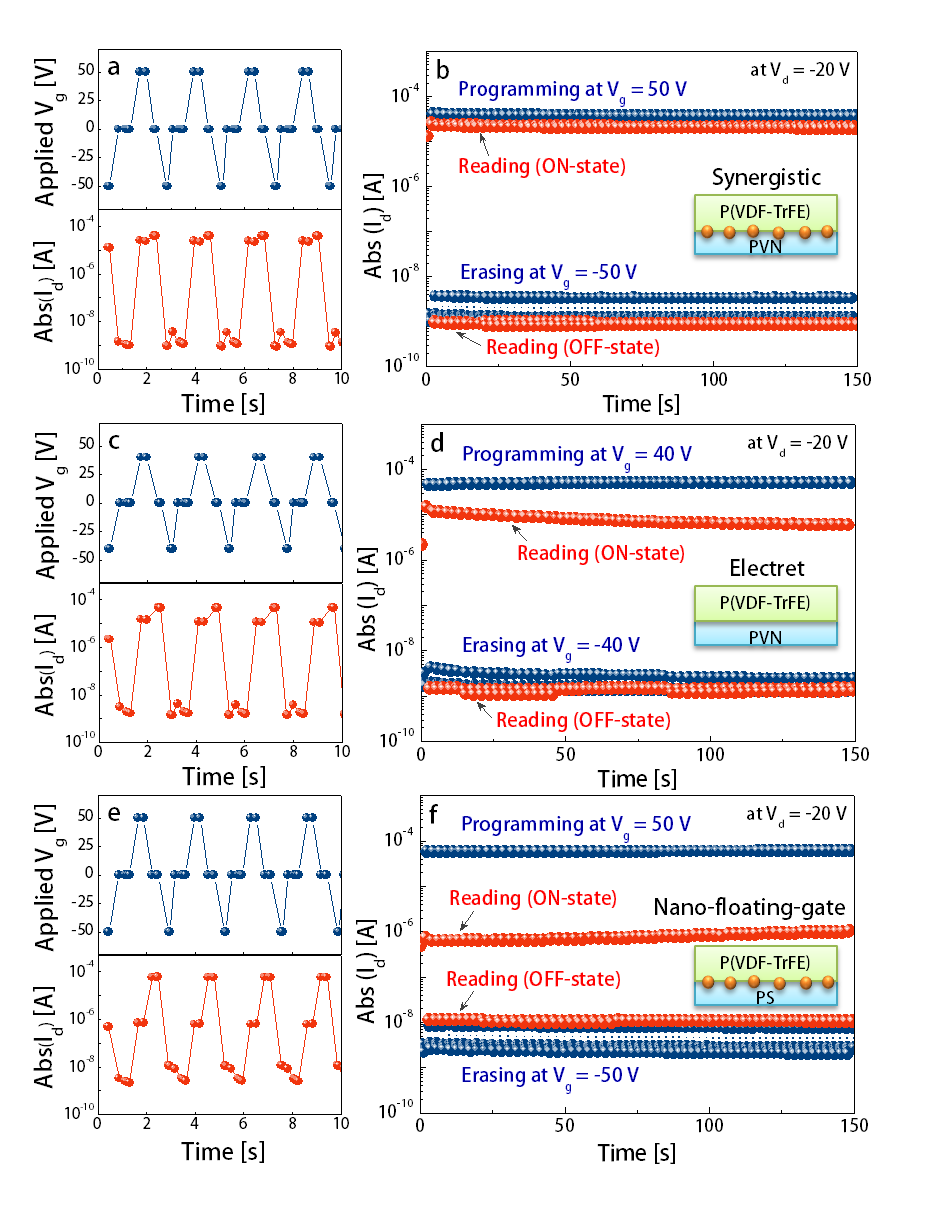


**Figure S4**. **Cycling endurance test**. The drain current (I_d_) were measured at V_d_ = = -20V and V_g_=0V after application of V_g_ = 50V and V_g_ = -50 for a,b) Synergistic memory and e, f) nano-floating-gate memory, and V_g_ = 40V and V_g_ = -40V for c, d) Electret memory.

**
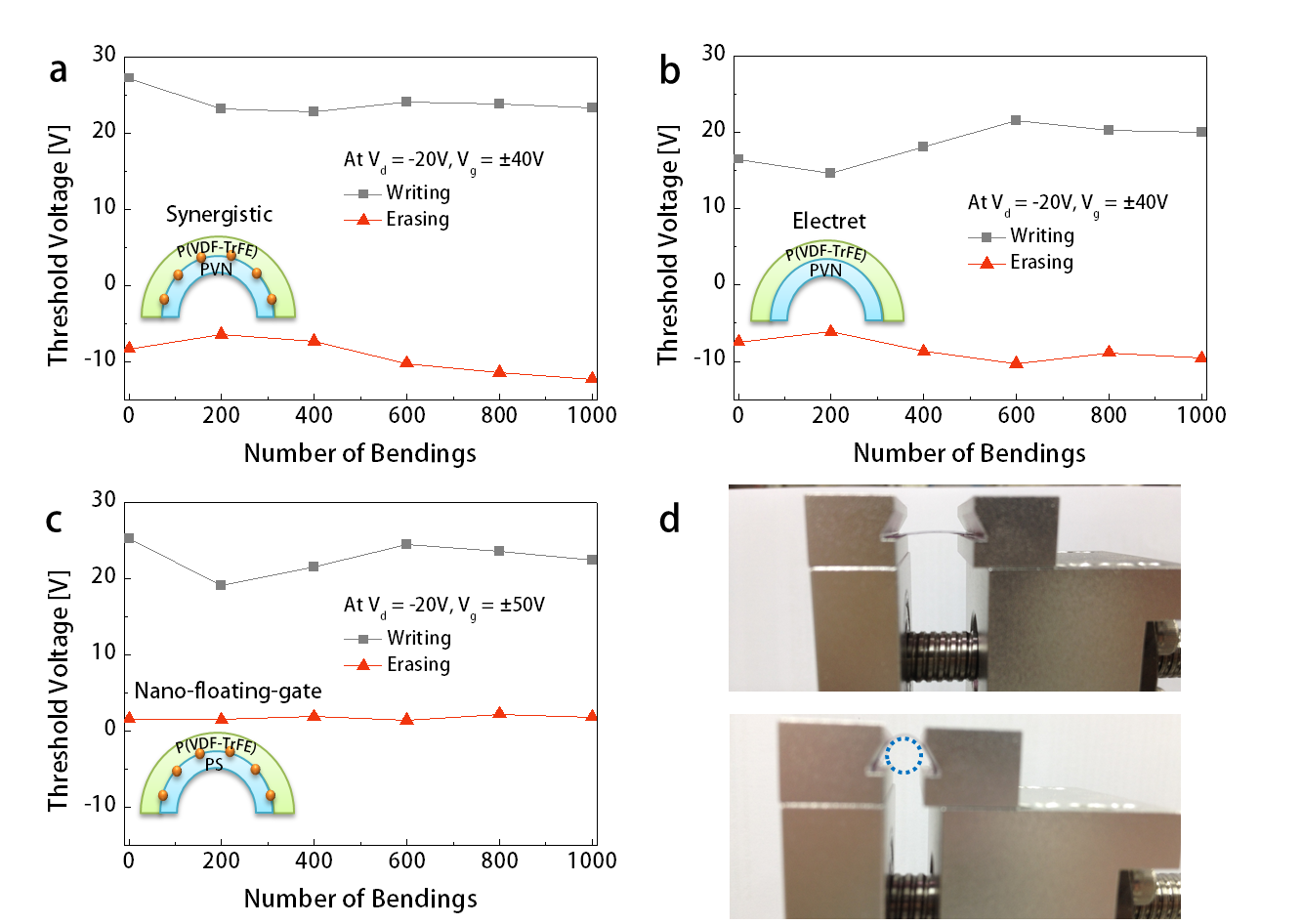
**

**Figure S5**. **Mechanical durability** during repeated bending at a bending radius of 3mm was measured in a) a SM, b) EM, and c) NFGM, respectively. Threshold voltages for both wiring and erasing states was plotted as a function of the number of bending cycles. d) Photographs show flexible OFET-type memories in bent and unbent status. The channel length and width of the memory devices were 20 and 1000 µm.
